# Risk factors for prognosis in patients with traumatic brain injury: A retrospective observational study

**DOI:** 10.1097/MD.0000000000048233

**Published:** 2026-04-10

**Authors:** Siya Meng, Linna He, Binghan Wang, Haigang Zhang, Mei Li, Jingguo Zhai

**Affiliations:** a Department of Critical Care Medicine, Shenzhen Nanshan People’s Hospital, Shenzhen, China; b College of Nursing, Southern Medical University, Guangzhou, China.

**Keywords:** coagulation disorders, prognosis, progressive hemorrhagic injury, risk factor, traumatic brain injury

## Abstract

To explore independent risk factors for early prognosis prediction in patients with traumatic brain injury (TBI). Retrospective observational study. Trauma center of a tertiary care hospital in Shenzhen, Guangdong, China. A total of 321 patients with TBI from January 2020 to December 2022. To identify risk factors that could predict the prognosis of patients with TBI, and explore the relationship between coagulation function, progressive hemorrhagic injury (PHI), and prognosis. Baseline characteristics, coagulation function-related measurements at admission, and occurrence of PHI were retrospectively obtained from medical records. Patient prognosis was evaluated by Glasgow outcome scale (GOS) score 1 month after hospital admission. Bivariate and multivariate logistic regression analyses were performed to identify risk factors associated with patient prognosis. The receiver operating characteristic curve analysis was applied to evaluate the diagnostic potential of each risk factor. There were 162 patients with good prognosis (GOS 4–5) and 159 patients with poor prognosis (GOS 1–3). Bivariate and multivariate logistic regression analyses showed the development of PHI (odds ratio [OR] = 11.154, 95% confidence interval [CI]: 4.926–25.260), brain herniation (OR = 8.588, 95% CI: 3.510–21.014), coagulation disorders (OR = 7.961, 95% CI: 3.676–17.242); history of blood transfusion within 24 hours of admission (OR = 3.474, 95% CI: 1.517–7.953); multiple injuries (OR = 3.277, 95% CI: 1.453–7.392); fluid replacement volumes within 24 hours of admission (OR = 2.881, 95% CI: 1.367–6.072); and Glasgow coma scale scores (OR = 2.523, 95% CI: 1.414–4.501) to be independent risk factors for poor prognosis. PHI had the highest area under the curve in receiver operating characteristic analysis. The occurrence of PHI, brain herniation, coagulation disorders, history of blood transfusion within 24 hours of admission, multiple injuries, fluid replacement volumes ≥ 2000 mL within 24 hours of admission, and low Glasgow coma scale scores could predict poor short-term prognosis in TBI patients.

## 1. Introduction

Traumatic brain injury (TBI) refers to the interruption of brain function, or other signs of brain pathology, owing to external physical impact.^[1]^ The annual global incidence of TBI was estimated at 50 million cases and approximately half of the global population is expected to experience TBI in their lifetime.^[2]^ TBI is a leading cause of death and disability in the population aged younger than 40 years, and is associated with greater morbidity and mortality in low- and middle-income nations.^[2]^ TBI cost the global economy ~400 billion United States dollars annually, representing 0.5% of the gross world product.^[3]^ In China, ~13 in 1,00,000 individuals died from TBI, and the mortality in severe cases approached 27%.^[4]^ TBI has the highest incidence among all common neurological diseases, and therefore represents a significant public health burden.^[1]^

Many studies have attempted to identify predictors of poor outcomes in patients following TBI. Old age, low Glasgow coma scale (GCS) scores at admission, high head abbreviated injury scale score, poor pupillary reflex, hypoxia, increased intracranial pressure, and need for tracheotomy were reported to be related with poor functional outcomes in these patients.^[5-7]^ Additionally, a high white blood cell count, high neutrophil lymphocyte ratio, anemia, hyperglycemia, high sodium level, and high uric acid level were also found to be closely related to unfavorable neurological prognoses in TBI patients.^[8-11]^

Coagulopathy and progressive hemorrhagic injury (PHI) were found to be important factors for secondary injury in TBI.^[12]^ The incidence of coagulopathy ranged from 7% to 97.5%, with the mortality rate up to 86% in TBI patients with coagulation disorders.^[13,14]^ In addition, patients with coagulation disorders had a 9-fold higher mortality rate and 30-fold higher risk of adverse outcomes than those without coagulopathy.^[15]^ Studies have shown that 21% to 64% of patients with TBI developed PHI, which was associated with a 5-fold higher risk of clinical deterioration and was a major cause of disability and death in these patients.^[16-18]^ Therefore, it is important to identify TBI with these high-risk factors for poor prognosis in order to facilitate early targeted management.

In this retrospective observational study, we collected and analyzed the clinical data of TBI patients, with the objective to identify risk factors that affect the early prognosis of patients with TBI, and explore the relationship between coagulation function, PHI, and prognosis.

## 2. Materials and methods

### 2.1. Study design and participant selection

We performed a retrospective observational study and reviewed patients with TBI and admitted into Shenzhen Nanshan People’s Hospital at Shenzhen, China, between January 2020 and December 2022. This hospital is a tertiary care hospital with a trauma center. The study was approved by the ethics committee of the Huazhong University of Science and Technology Union Shenzhen Hospital, Shenzhen, China (approval number: KY-2023-092701). The informed consent was waived due to the retrospective study design.

The inclusion criteria were patients aged ≥18 years, brain injury with 24 hours before hospital admission, diagnosed with TBI based on computed tomography (CT) or magnetic resonance imaging that were performed at hospital admission and a repeat scan within 72 hours after hospitalization. The exclusion criteria were patients with a history of coagulation disorders or systemic illnesses, brain diseases (traumatic or non-traumatic) with permanent neurological deficits, and autoimmune diseases, severe organ damage or failure, or spinal cord injuries. In addition, patients with incomplete medical records and women who were menstruating, pregnant, or breastfeeding were also excluded.

### 2.2. Clinical information and imaging result collection

Medical records were reviewed to retrieve patient baseline information and clinical characteristics. The Glasgow outcome scale (GOS) scoring system was used to assess the prognosis of TBI patients 1 month after the hospital admission.^[19]^ The assessment scores were as follows: 5 (good recovery, but with minor defects), 4 (mild disability, but able to live independently), 3 (conscious, but unable to live independently due to severe disability), 2 (vegetative state), and 1 (death). Among these, scores of 4 to 5 and 1 to 3 were considered to be associated with good and poor prognosis, respectively. Patients were categorized into good and poor prognosis groups based on GOS score accordingly.

PHI was defined as an increase in the initial lesion size by at least 25% based on the comparison between initial admission CT scan with the 72-hour follow-up CT scan, or the appearance of new hemorrhagic lesions that were not detected on first brain CT.^[20]^

### 2.3. Laboratory test result collection

Following hospital admission, 5 mL of peripheral venous blood was obtained for laboratory tests. The prothrombin time (PT), activated partial thromboplastin time (APTT), fibrinogen (FIB) level, and international normalized ratio (INR) were measured using the STA-R Max analyzer (Stago, USA). The platelet count (PLT) was evaluated using the XN9000 analyzer (SYSMEX, Japan). The reference values for the evaluated indicators were: PT 11.0 to 14.5 seconds, APTT 28.0 to 43.0 seconds, FIB 2.0 to 4.22 g/L, INR 0.85 to 1.15, and PLT 125 to 350 × 10^9^/L.

The disseminated intravascular coagulation score, formulated by the International Society of Thrombosis and Haemostasis in 2001,^[21]^ was used to evaluate coagulation function. Disseminated intravascular coagulation score consists of PLT, PT, FIB, and elevated fibrin-related marker, and was assessed 24 hours after hospital admission, with a score ≥5 indicating coagulation dysfunction and a score <5 suggesting normal coagulation function.

### 2.4. Statistical analysis

Data analysis was performed in SPSS 22.0 (IBM). Normally distributed continuous data were presented as means ± standard deviation, and compared by *t*-tests and 1-way analysis of variance. To evaluate homogeneity of variance, the Bonferroni’s test was used for pairwise comparisons between groups and the Tamhane’s test was used for heterogeneity of variance. Continuous data not conforming to a normal distribution were presented as median with interquartile range (IQR), and compared using the Mann–Whitney *U* test. Categorical data were presented as frequencies and/or percentages and compared using the Chi-square test. Spearman’s rank correlation analysis was used to evaluate the correlation between risk factors and prognosis. The collinearity was considered to be absent if the tolerance was >0.1 with the variance inflation factor <5. To identify the risk factors that affected prognosis in patients with TBI, multivariate logistic regression analysis was performed using the variables that demonstrated strong correlation. A receiver operating characteristic (ROC) curve was created from independent risk factors identified from the multivariate logistic regression analysis. The model’s discriminatory power was evaluated by calculating the area under the curve (AUC) of the ROC curve. A *P* < .05 was considered statistically significant.

## 3. Results

### 3.1. Baseline characteristics of study participants

A total of 321 patients with TBI were included in the current study. There were 162 (138 male and 24 female) patients with an average age of 51.34 (IQR 35.00, 65.00) years old were included in the good prognosis group. The poor prognosis group included 159 (129 male and 30 female) patients with an average age of 55.30 (IQR 40.00, 72.00) years old.

Bivariate analysis of baseline characteristics between 2 groups of patients demonstrated statistically significant differences in terms of the presence of brain herniation, community-acquired pneumonia, and coagulation disorders, GCS scores, fluid replacement volume within 24 hours of admission, history of blood transfusion within 24 hours of admission, surgery within 24 hours of admission, cerebral contusion and laceration, smoking, and liver disease, presence of multiple injurie, mean arterial pressure at admission, and the pH value (Table 1). There were no statistically significant differences in other measurements.

**Table 1:** Comparison between 2 groups with different prognosis.

Characteristics	Good prognosis group (n = 162)	Poor prognosis group (n = 159)	*χ* ^*2*^ */Z*	*P*
Sex			0.942	.332
Male	138 (85.19)	129 (81.13)		
Female	24 (14.81)	30 (18.87)		
Age (yr)	51.34 (35.00, 65.00)	55.30 (40.00, 72.00)	−1.457	.145
Chronic alcohol drinking	27 (16.67)	21 (13.21)	0.755	.385
Smoking history	36 (22.22)	15 (9.43)	7.547	.006
History of long-term anticoagulant use	2 (1.23)	156 (98.11)	3.085	.079
History of liver disease	13 (8.02)	46 (28.93)	23.378	<.001
Coagulation disorders	66 (40.74)	99 (62.26)	14.881	<.001
Community-acquired pneumonia	60 (37.04)	114 (71.70)	38.836	<.001
Body temperature at admission (°C)	36.60 (36.50, 36.80)	36.70 (36.50, 36.80)	−0.540	.589
MAP at admission (mm Hg)	100.60 (92.00, 111.00)	105.50 (92.00, 120.00)	−2.177	.029
GCS score at admission			178.369	<.001
13–15	106 (65.43)	2 (1.26)		
9–12	54 (33.33)	77 (48.43)		
3–8	2 (1.24)	80 (50.31)		
Antibiotic use within 24 h of injury	114 (70.37)	129 (81.13)	0.181	.670
Fluid replacement volume within 24 h of admission			26.924	<.001
<2000 mL	108 (66.67)	60 (37.74)		
≥2000 mL	54 (33.33)	99 (62.26)		
Blood transfusion within 24 h of admission	24 (14.81)	84 (52.83)	51.944	<.001
Surgery within 24 h of admission	75 (46.30)	111 (69.81)	18.208	<.001
Brain herniation	15 (9.26)	84 (52.83)	71.420	<.001
Diffuse axonal damage	6 (3.70)	12 (7.55)	2.239	.135
Cerebral contusion and laceration	57 (35.19)	99 (62.26)	23.554	<.001
Skull base fracture	60 (37.04)	63 (39.62)	0.227	.634
Multiple injuries	114 (70.37)	129 (81.13)	5.052	.025
Blood pH at admission	7.45 (7.44, 7.50)	7.44 (7.40, 7.49)	−1.921	.055

Data are presented as median (interquartile range) or number (%).

GCS = Glasgow coma scale, MAP = mean arterial pressure.

### 3.2. Comparison of coagulation function between patients with different prognosis

As shown in Table 2, the APTT, PT, INR, FIB, and PLT values at admission differed significantly between TBI patients with good or poor prognosis (all *P* < .05).

**Table 2:** Comparisons of coagulation function at admission between groups with different prognoses.

Group	APTT (s)	PT (s)	INR	FIB (g/L)	PLT (×10^9^/L)
Poor prognosis group (n = 159)	37.55 (35.00, 40.23)	15.07 (13.00, 14.80)	1.27 (0.99, 1.18)	2.72 (1.90, 3.20)	225.70 (166.00, 257.00)
Good prognosis group (n = 162)	36.34 (34.30, 37.20)	13.38 (12.90, 13.80)	1.05 (0.98, 1.12)	2.91 (2.13, 3.14)	233.98 (177.00, 288.00)
*Z*	−3.278	−3.653	−2.893	−2.842	−1.997
*P*	.001	<.001	.004	.004	.046

Data are presented as median (interquartile range).

APTT = activated partial thromboplastin time, FIB = fibrinogen, INR = international normalized ratio, PLT = platelet count, PT = prothrombin time.

### 3.3. Comparison of prognosis between patients with and without PHI

There were statistically significant differences between the PHI group and the non-PHI group in terms of APTT, PT, and INR at admission (*P* < .05; Table 3). Patients with and without PHI showed a statistically significant difference in terms of prognosis (*P* < .05; Table 4).

**Table 3:** Comparisons of coagulation function at admission between groups with and without PHI.

Group	APTT (s)	PT (s)	INR	FIB (g/L)	PLT (×10^9^/L)
Non-PHI (n = 139)	35.68 (31.90, 37.90)	13.60 (12.90, 14.30)	1.07 (0.99, 1.13)	2.82 (2.10, 3.30)	233.65 (177.00, 286.00)
PHI (n = 182)	37.20 (30.30, 40.80)	15.34 (12.90, 14.63)	1.22 (0.98, 1.16)	2.82 (2.09, 3.05)	226.99 (168.25, 275.00)
*Z*	−3.238	−3.932	−4.324	−0.594	−1.193
*P*	.001	.000	.000	.553	.233

Data are presented as median (interquartile range).

APTT = activated partial thromboplastin time, FIB = fibrinogen, INR = international normalized ratio, PHI = progressive hemorrhagic injury, PLT = platelet count, PT = prothrombin time.

**Table 4:** Comparisons of prognosis between groups with and without PHI.

Group	GOS (1 point)	GOS (2 points)	GOS (3 points)	GOS (4 points)	GOS (5 points)	Good prognosis	Poor prognosis	*χ* ^*2*^	*P*
Non-PHI (n = 139)	9 (6.47)	1 (0.72)	13 (9.35)	26 (18.71)	90 (64.75)	116 (83.45)	23 (16.55)	115.503	<.001
PHI (n = 182)	42 (23.08)	5 (2.74)	89 (48.90)	22 (12.09)	24 (13.19)	46 (25.27)	136 (74.73)		

Data are presented as number (%).

GOS = Glasgow outcome score, PHI = progressive hemorrhagic injury.

### 3.4. Correlation analysis

Spearman rank correlation analysis demonstrated positive correlation between prognosis and the development of brain herniation (*r* = 0.472, *P* < .001), community-acquired pulmonary infections (*r* = 0.348, *P* < .001), coagulation disorders (*r* = 0.215, *P* < .001), GCS scores (*r* = 0.316, *P* < .001), fluid replacement volume within 24 hours of admission (*r* = 0.290, *P* < .001), history of blood transfusion within 24 hours of admission (*r* = 0.402, *P* < .001), surgery within 24 hours of admission (*r* = 0.238, *P* < .001), and cerebral contusion and laceration (*r* = 0.271, *P* < .001), presence of multiple injuries (*r* = 0.125, *P* = .025), PHI (*r* = 0.577, *P* < .001), APTT (*r* = 0.183, *P* = .001), PT (*r* = 0.264, *P* < .001), and INR (*r* = 0.282, *P* < .001). The values of FIB correlated negatively with prognosis (*r* = −0.226, *P *< .001). No correlation was found between prognosis and smoking history, history of liver disease, mean arterial pressure at admission, and PLT counts (all *P* > .05).

### 3.5. Multicollinearity tests

Multicollinearity tests were performed on 9 factors that demonstrated statistical significance in both bivariate and correlation analyses. Results indicated variance inflation factor < 5 and tolerance values > 0.1 for all variables, suggesting absence of multicollinearity (Table 5).

**Table 5:** Multicollinearity diagnostics.

Variables	VIF	Tolerance
PHI	1.401	0.714
Brain herniation	1.402	0.713
Coagulation disorders	1.323	0.756
History of blood transfusion within 24 h of admission	1.341	0.746
Multiple injuries	1.035	0.966
Fluid replacement volume within 24 h of admission	1.169	0.856
GCS scores	1.088	0.919
Surgery within 24 h of admission	1.022	0.978
Cerebral contusion and laceration	1.054	0.949

GCS = Glasgow coma scale, PHI = progressive hemorrhagic injury, VIF = variance inflation factor.

### 3.6. Multivariable logistic regression analysis

The above 9 variables that correlated with prognosis were included for a multivariate logistic regression analysis. The presence of PHI, coagulation disorders, brain herniation, multiple injuries, GCS scores, history of blood transfusion within 24 hours of admission, and fluid replacement volumes within 24 hours of admission were found to be independent risk factors for prognosis in these patients (Table 6).

**Table 6:** Multivariate logistic regression analysis of variables associated with prognosis in patients with TBI.

Variables	β	SE	Wald	*P*	OR	95% CI
PHI	2.412	0.417	33.445	<.001	11.154	4.926–25.260
Brain herniation	2.150	0.457	22.183	<.001	8.588	3.510–21.014
Coagulation disorders	2.075	0.394	27.680	<.001	7.961	3.676–17.242
History of blood transfusion within 24 h of admission	1.245	0.423	8.681	.003	3.474	1.517–7.953
Multiple injuries	1.187	0.415	8.179	.004	3.277	1.453–7.392
Fluid replacement volume within 24 h of admission	1.058	0.380	7.736	.005	2.881	1.367–6.072
GCS scores	0.925	0.295	9.814	.002	2.523	1.414–4.501

CI = confidence interval, GCS = Glasgow coma scale, OR = odds ratio, PHI = progressive hemorrhagic injury, SE = standard error, TBI = traumatic brain injury.

### 3.7. ROC curve analysis

**Figure 1.**
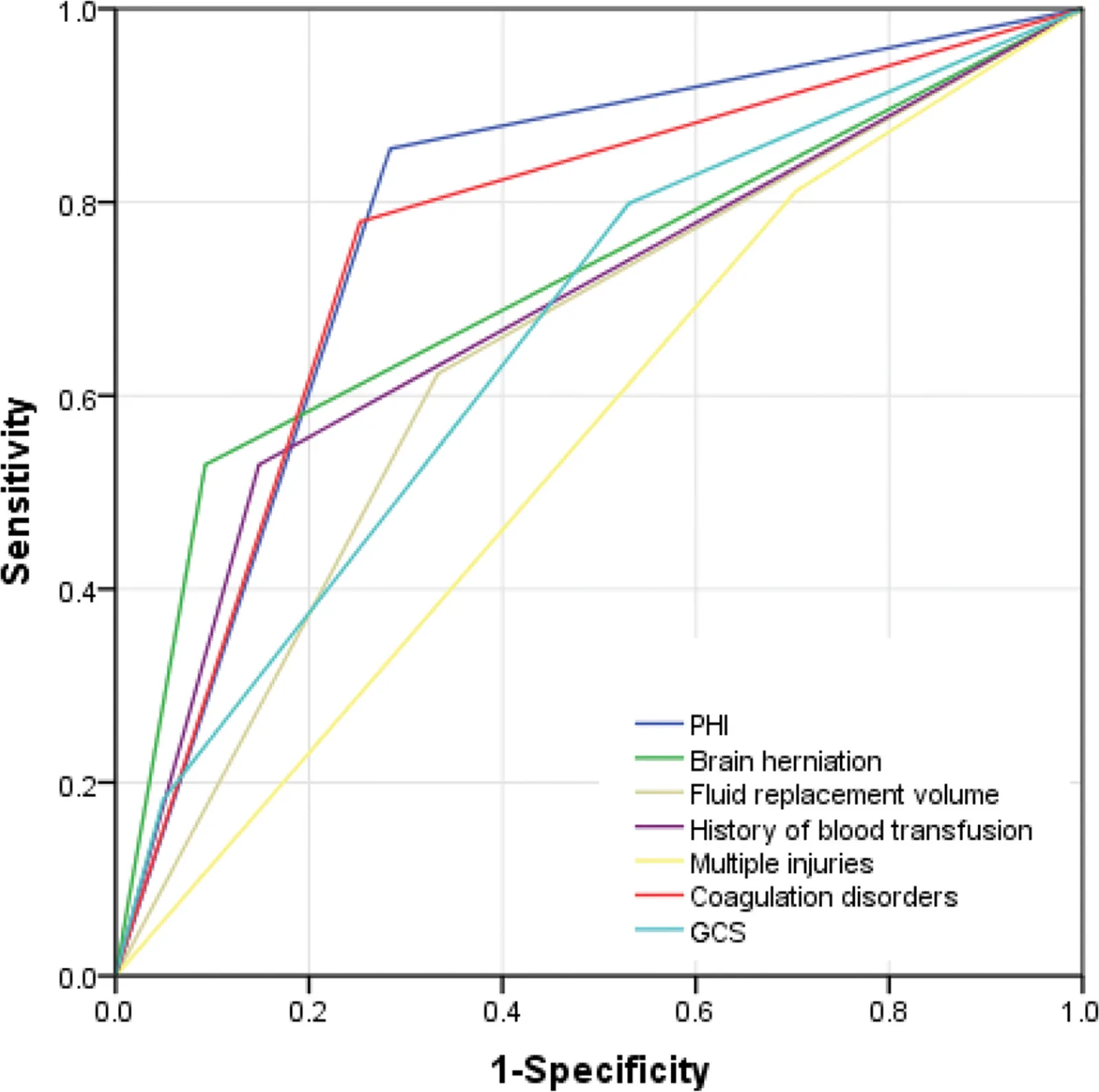
ROC analyses of the 7 independent risk factors obtained from the multivariate logistic regression analysis. GCS = Glasgow coma scale, PHI = progressive hemorrhagic injury, ROC = receiver operating characteristic.

The independent risk factors obtained from the multivariate logistic regression model were entered into the ROC analyses (Fig. 1). Table 7 presents the measurements in the ROC curves of the 7 independent risk factors. Among these factors, PHI had the highest AUC to predict patient prognosis.

**Table 7:** ROC curve analyses of 7 independent risk factors for prognosis in TBI patients.

Variables	AUC	SE	95% CI	Cut-off value
PHI	0.786	0.026	0.734–0.838	NA
Brain herniation	0.718	0.029	0.661–0.775	NA
Coagulation disorders	0.763	0.027	0.710–0.817	NA
History of blood transfusion within 24 h of admission	0.690	0.030	0.631–0.749	NA
Multiple injuries	0.554	0.032	0.491–0.617	NA
Fluid replacement volume within 24 h of admission	0.645	0.031	0.584–0.705	NA
GCS scores	0.663	0.030	0.604–0.722	NA

AUC = area under the curve, CI = confidence interval, GCS = Glasgow coma scale, NA = not available, ROC = receiver operating characteristic, SE = standard error, TBI = traumatic brain injury.

## 4. Discussion

TBI not only affects individual patients but also impacts their families and society. Ealy identification of TBI patients with poor prognosis could facilitate targeted therapy. Here, we showed that the occurrence of PHI, brain herniation, coagulation disorders, history of blood transfusion within 24 hours of admission, multiple injuries, fluid replacement volume within 24 hours of admission and GCS scores were independent risk factors for prognosis in patients with TBI. In particular, PHI, brain herniation and coagulation disorders were found to have notable impact on poor prognosis in these patients.

Coagulation disorders represent a common complication in patients with TBI. After injury, the intrinsic and extrinsic coagulation pathways are activated, with subsequent release of catecholamines and corticosteroids which in turn exacerbates the systemic coagulation impairments.^[22]^ Coagulation disorders primarily involve the development of a hypercoagulable state following trauma and secondary hyperfibrinolysis. This considerably increases the risk of rebleeding and death and has a great impact on patient prognosis.^[23]^ In our study, 51.40% of patients had coagulation disorders, and 62.26% of them demonstrated poor prognosis. The APTT, PT, and INR values at admission were all lower in the group with good prognosis than in that with poor prognosis, despite that the values of FIB and PLT were significantly higher in the former group. Coagulation disorders represented an independent risk factor for prognosis in patients with TBI. This was consistent with findings from other studies.^[12,24]^ PT, APTT, and FIB are commonly used indicators of coagulation. PT and APTT reflect the status of the extrinsic and intrinsic coagulation systems, respectively. FIB values are found to decrease in TBI due to excessive consumption. Studies on patients with severe injuries have shown that 17% of them demonstrated significant reductions in thrombin production upon hospital admission.^[25]^ Low FIB levels at admission was found to be independently associated with increased injury severity and shock. In addition, the FIB level at admission was found to be an independent predictor of 24-hour and 28-day mortality.^[26]^ In cases of major trauma, the main factors contributing to hypofibrinogenemia include hemodilution (due to fluid resuscitation), blood loss, consumption due to clot formation at the site of injury, hypothermia (impairing FIB synthesis), fibrinogenolysis, and increased degradation owing to acidosis.^[26]^ Thrombocytopenia is a marker of poor prognosis following injury, and is independently associated with a history of blood transfusion, progression of brain injury, and post-injury mortality.^[27]^ Another study showed that PLT between 0 and 100 × 10^9^/L, 101 to 200 × 10^9^/L, 201 to 300 × 10^9^/L, and ≥301 × 10^9^/L at admission were associated with a 24-hour mortality rate of 33%, 30%, 21%, and 14%, respectively. The probability of death within 24 hours decreased with an increase in PLT, indicating a correlation between decreased PLT and increased mortality rates.^[28]^ Coagulation disorders represent an independent risk factor for poor prognosis in these patients. It is therefore essential to monitor coagulation changes when TBI patients were admitted into hospital, as this may help personalized management, especially intensive monitoring and treatment in patients with predicted poor prognosis.

PHI is caused by secondary hemorrhage and could develop in ~55.6% of cases.^[29]^ PHI is a major cause of death and disability in patients with TBI. Research has shown that PHI is strongly associated with poor prognosis in patients with TBI.^[29]^ Coagulation disorders represent one of the primary mechanisms that lead to the development of PHI following TBI. Local consumptive coagulopathy and excessive fibrinolysis following TBI can lead to recurrent bleeding and persistent enlargement of subdural hematomas. The mass effect of hematomas can cause a series of fatal complications, such as increased intracranial pressure, brain herniation, and respiratory depression, all contributing to poor prognosis. However, the biochemical markers associated with coagulation disorders and PHI have been reportedly inconsistently. Stein et al found that abnormalities in INR, APTT, and PLT values correlated independently with the development of PHI.^[30]^ Zhang et al demonstrated a positive correlation between PT, d-dimer, and INR values and the risk of developing PHI.^[31]^ Yuan et al showed significant correlation between INR and PLT values and PHI.^[32]^ Juratli et al reported a 47.1% prevalence of coagulopathy in a prospective study on TBI patients.^[33]^ Among these patients, 43.5% showed early hemorrhagic progression of cerebral contusions within the first 6 hours. Notably, PLT and FIB values were independent predictors for PHI. In our study, there were statistically significant differences in the comparison of APTT, PT, and INR at admission between the PHI group and the non-PHI group. The incidence rate of PHI was found to be 43.0%, and 85.5% of patients who developed PHI had a poor prognosis. The groups with and without PHI demonstrated a statistically significant different prognosis. In addition, the development of PHI correlated positively with patient prognosis and demonstrated significant impact on the prognosis in TBI patients. Among all the tested indicators, PHI had the highest AUC, suggesting that PHI was more accurate than the other independent indicators to predict the prognosis in TBI patients. This finding is similar to the reports from other studies.^[20,34]^ Patients with TBI often present with evident symptoms immediately or within a few hours after the injury. However, early symptoms of PHI may be mild and difficult to detect. This leads to delays in clinical diagnosis and treatment, substantially increasing the mortality rate and adverse outcomes in patients with TBI. Therefore, it is essential to closely monitor them for the development of PHI. Further high-quality studies are needed to identify variations in coagulation indicators among patients with TBI who develop PHI.

In addition to the risk factors described above, low GCS scores, brain herniation, multiple injuries, history of blood transfusion within 24 hours of admission, and fluid replacement volumes of ≥2000 mL within 24 hours of admission were found to be independent risk factors for prognosis in patients with TBI. Low GCS scores and the presence of brain herniation correlated with poor prognosis in these patients. GCS score is commonly used for evaluating the severity of TBI. Previous studies suggested that lower GCS scores at admission were associated with higher mortality rates.^[35,36]^ GCS score was considered to be an effective predictor of prognosis in TBI.^[34]^ Our findings indicated concurrent brain herniation to be an independent risk factor for poor prognosis in TBI patients. Brain herniation predisposes to posttraumatic cerebral infarction in TBI, which in turn has considerable impact on patient prognosis.^[37]^ Allison et al suggested that early blood transfusion could replenish coagulation factors and reduce the occurrence of progressive hemorrhage.^[38]^ However, Vedantam et al found that a higher transfusion threshold increases the risk of PHI in patients with severe TBI.^[39]^ Notably, patients with transfusion thresholds exceeding 10 g/dL have a 2.3-fold higher risk of developing PHI. Frequent and excessive blood transfusion may lead to microvascular damage and trigger PHI. Blood transfusion thresholds therefore need to be stringently maintained in the clinical practice.^[40]^

Patients with TBI who have multiple injuries are usually seriously ill and experience a higher incidence of complications and mortality. Their treatment is therefore a considerable clinical challenge. Severe TBI is often accompanied by variable degrees of shock, leading to systemic pathophysiological changes. Although active fluid resuscitation can correct hypothermia and acidosis, it may dilute coagulation factors and result in severe coagulation disorders.^[41]^ Based on their findings, Chang et al recommended the use of crystalloid solutions for resuscitation (> 2000 mL) in patients with TBI who are at increased risk of mortality.^[42]^ Restrictive fluid resuscitation therefore needs to be initiated early to ensure adequate cerebral perfusion, prevent a relative decrease in coagulation factors consequent to hemodilution, and reduce the complications associated with TBI. In conjunction, these management may help improve patient prognosis.

Our study has certain limitations. First, this was a single-center retrospective clinical study, which might have significant selection bias to impact the study results. Second, data pertaining to thromboelastography parameters could not be obtained in this study owing to various constraints, hindering the accurate diagnosis of platelet disorders, fibrinogen deficiency, and hyperfibrinolysis. In addition, we collected the laboratory test results at the hospital admission but not after admission, since the in-hospital laboratory tests were collected at various timepoints. A longitudinal trend of the test result changes might provide more accurate prognosis prediction, which requires further studies. Finally, due to limitations in the original medical records, we were able to collect the GOS score only until 1 month after admission as the outcome variable. Long-term prognosis of patients after discharge should be collected as further evaluation criteria. Further studies including larger sample sizes and appropriate durations of follow-up are needed to validate our findings and provide stronger evidence for predicting patient outcomes.

## 5. Conclusions

The occurrence of PHI, brain herniation, coagulation disorders, history of blood transfusion within 24 hours of admission, multiple injuries, fluid replacement volumes of ≥2000 mL within 24 hours of admission, and low GCS scores were risk factors for poor prognosis in patients with TBI. In particular, the occurrence of PHI and coagulation disorders both had significant impacts on the prognosis of these patients. It is therefore essential to closely monitor coagulation indicators in TBI patients, which could help assess the course of disease progression and prognosis. Among the modifiable risk factors, the blood transfusion threshold within 24 hours of admission and the volume of early fluid resuscitation could impact coagulation function and should be managed carefully to ameliorate the development of PHI in patients with TBI and improve their prognosis.

## Author contributions

**Conceptualization:** Jingguo Zhai.

**Data curation:** Binghan Wang, Jingguo Zhai.

**Formal analysis:** Linna He, Binghan Wang, Jingguo Zhai.

**Funding acquisition:** Siya Meng, Haigang Zhang.

**Investigation:** Siya Meng, Binghan Wang.

**Methodology:** Siya Meng, Haigang Zhang.

**Supervision:** Haigang Zhang, Mei Li.

**Validation:** Mei Li.

**Writing – original draft:** Siya Meng.

**Writing – review & editing:** Linna He, Mei Li, Jingguo Zhai.

## References

[1] Capizzi A Woo J Verduzco-Gutierrez M. Traumatic brain injury: an overview of epidemiology, pathophysiology, and medical management. Med Clin North Am. 2020;104:213–38. 10.1016/j.mcna.2019.11.001 32035565

[2] GBD 2016 Neurology Collaborators. Global, regional, and national burden of neurological disorders, 1990-2016: a systematic analysis for the Global Burden of Disease Study 2016. Lancet Neurol. 2019;18:459–80. 10.1016/S1474-4422(18)30499-X PMC6459001 30879893

[3] Maas AIR Menon DK Manley GT. Traumatic brain injury: progress and challenges in prevention, clinical care, and research. Lancet Neurol. 2022;21:1004–60. 10.1016/S1474-4422(22)00309-X PMC10427240 36183712

[4] Jiang JY Gao GY Feng JF. Traumatic brain injury in China. Lancet Neurol. 2019;18:286–95. 10.1016/S1474-4422(18)30469-1 30784557

[5] Tian R Liu W Dong J. Prognostic predictors of early outcomes and discharge status of patients undergoing decompressive craniectomy after severe traumatic brain injury. World Neurosurg. 2019;126:e101–8. 10.1016/j.wneu.2019.01.246 30790726

[6] Khalili H Paydar S Safari R Arasteh P Niakan A Foroughi A A. Experience with traumatic brain injury: is early tracheostomy associated with better prognosis? World Neurosurg. 2017;103:88–93. 10.1016/j.wneu.2017.02.060 28254541

[7] Lenell S Nyholm L Lewén A Enblad P. Clinical outcome and prognostic factors in elderly traumatic brain injury patients receiving neurointensive care. Acta Neurochir (Wien). 2019;161:1243–54. 10.1007/s00701-019-03893-6 PMC6525667 30980243

[8] Chen J Qu X Li Z Zhang D Hou L. Peak neutrophil-to-lymphocyte ratio correlates with clinical outcomes in patients with severe traumatic brain injury. Neurocrit Care. 2019;30:334–9. 10.1007/s12028-018-0622-9 30288677

[9] Li Z Wu X Wu X. Admission circulating monocytes level is an independent predictor of outcome in traumatic brain injury. Brain Inj. 2018;32:515–22. 10.1080/02699052.2018.1429023 29355430

[10] Dolmans RGF Hulsbergen AFC Gormley WB Broekman MLD. Routine blood tests for severe traumatic brain injury: can they predict outcomes? World Neurosurg. 2020;136:e60–7. 10.1016/j.wneu.2019.10.086 31655234

[11] Liu H He J Zhong J. Clinical and basic evaluation of the prognostic value of uric acid in traumatic brain injury. Int J Med Sci. 2018;15:1072–82. 10.7150/ijms.25799 PMC6036155 30013449

[12] Maegele M Schöchl H Menovsky T. Coagulopathy and haemorrhagic progression in traumatic brain injury: advances in mechanisms, diagnosis, and management. Lancet Neurol. 2017;16:630–47. 10.1016/S1474-4422(17)30197-7 28721927

[13] Nakae R Murai Y Morita A Yokobori S. Coagulopathy and traumatic brain injury: overview of new diagnostic and therapeutic strategies. Neurol Med Chir (Tokyo). 2022;62:261–9. 10.2176/jns-nmc.2022-0018 PMC9259082 35466118

[14] Epstein DS Mitra B Cameron PA Fitzgerald M Rosenfeld JV. Acute traumatic coagulopathy in the setting of isolated traumatic brain injury: Definition, incidence and outcomes. Br J Neurosurg. 2015;29:118–22. 10.3109/02688697.2014.950632 25153987

[15] Böhm JK Güting H Thorn S. Global Characterisation of Coagulopathy in Isolated Traumatic Brain Injury (iTBI): a CENTER-TBI Analysis. Neurocrit Care. 2021;35:184–96. 10.1007/s12028-020-01151-7 PMC8285342 33306177

[16] Gulati G Iffland PH 2nd Janigro D Zhang B Luggen ME. Anti-NR2 antibodies, blood-brain barrier, and cognitive dysfunction. Clin Rheumatol. 2016;35:2989–97. 10.1007/s10067-016-3339-1 27357716

[17] Miles MVP Hicks RC Parmer H. Traumatic brain injury patients with platelet inhibition receiving platelet transfusion demonstrate decreased need for neurosurgical intervention and decreased mortality. J Trauma Acute Care Surg. 2022;92:701–7. 10.1097/TA.0000000000003516 35320155

[18] Guillotte AR Herbert JP Madsen R Hammer RD Litofsky NS. Effects of platelet dysfunction and platelet transfusion on outcomes in traumatic brain injury patients. Brain Inj. 2018;32:1849–57. 10.1080/02699052.2018.1536805 30346865

[19] McMillan T Wilson L Ponsford J Levin H Teasdale G Bond M. The Glasgow Outcome Scale - 40 years of application and refinement. Nat Rev Neurol. 2016;12:477–85. 10.1038/nrneurol.2016.89 27418377

[20] Wan X Fan T Wang S. Progressive hemorrhagic injury in patients with traumatic intracerebral hemorrhage: characteristics, risk factors and impact on management. Acta Neurochir (Wien). 2017;159:227–35. 10.1007/s00701-016-3043-6 27943076

[21] Yuh EL Jain S Sun X. Pathological computed tomography features associated with adverse outcomes after mild traumatic brain injury: a TRACK-TBI study with external validation in CENTER-TBI. JAMA Neurol. 2021;78:1137–48. 10.1001/jamaneurol.2021.2120 PMC8290344 34279565

[22] Pastor IS Para I Vesa C Florian I. Identifying predictive factors for mortality in patients with TBI at a neurosurgery department. J Med Life. 2023;16:554–8. 10.25122/jml-2023-0114 PMC10251389 37305827

[23] Maegele M Aversa J Marsee MK. Changes in Coagulation following Brain Injury. Semin Thromb Hemost. 2020;46:155–66. 10.1055/s-0040-1702178 32160642

[24] Meyfroidt G Bouzat P Casaer MP. Management of moderate to severe traumatic brain injury: an update for the intensivist. Intensive Care Med. 2022;48:649–66. 10.1007/s00134-022-06702-4 35595999

[25] von der Brelie C Schneegans I van den Boom L Meier U Hedderich J Lemcke J. Impaired coagulation is a risk factor for clinical and radiologic deterioration in patients with traumatic brain injury and isolated traumatic subarachnoid hemorrhage. J Trauma Acute Care Surg. 2015;79:295–300. 10.1097/TA.0000000000000722 26218700

[26] Moore EE Moore HB Kornblith LZ. Trauma-induced coagulopathy. Nat Rev Dis Primers. 2021;7:30. 10.1038/s41572-021-00264-3 PMC9107773 33927200

[27] Kornblith LZ Moore HB Cohen MJ. Trauma-induced coagulopathy: the past, present, and future. J Thromb Haemost. 2019;17:852–62. 10.1111/jth.14450 PMC6545123 30985957

[28] Kim H Jin ST Kim YW Kim SR Park IS Jo KW. Risk factors for early hemorrhagic progression after traumatic brain injury: a focus on lipid profile. J Neurotrauma. 2015;32:950–5. 10.1089/neu.2014.3697 25557755

[29] Zhang J Zhang F Dong JF. Coagulopathy induced by traumatic brain injury: systemic manifestation of a localized injury. Blood. 2018;131:2001–6. 10.1182/blood-2017-11-784108 PMC5934798 29507078

[30] Henriksen HH Grand AG Viggers S. Impact of blood products on platelet function in patients with traumatic injuries: a translational study. J Surg Res. 2017;214:154–61. 10.1016/j.jss.2017.02.037 28624038

[31] Zhang D Gong S Jin H. Coagulation parameters and risk of progressive hemorrhagic injury after traumatic brain injury: a systematic review and meta-analysis. Biomed Res Int. 2015;2015:261825. 10.1155/2015/261825 PMC4589576 26457298

[32] Yuan Q Sun YR Wu X. Coagulopathy in traumatic brain injury and its correlation with progressive hemorrhagic injury: a systematic review and meta-analysis. J Neurotrauma. 2016;33:1279–91. 10.1089/neu.2015.4205 26850305

[33] Juratli TA Zang B Litz RJ. Early hemorrhagic progression of traumatic brain contusions: frequency, correlation with coagulation disorders, and patient outcome: a prospective study. J Neurotrauma. 2014;31:1521–7. 10.1089/neu.2013.3241 24738836

[34] van Gent JAN van Essen TA Bos MHA Cannegieter SC van Dijck J Peul WC. Coagulopathy after hemorrhagic traumatic brain injury, an observational study of the incidence and prognosis. Acta Neurochir (Wien). 2020;162:329–36. 10.1007/s00701-019-04111-z PMC6982633 31741112

[35] Gao G Wu X Feng J. Clinical characteristics and outcomes in patients with traumatic brain injury in China: a prospective, multicentre, longitudinal, observational study. Lancet Neurol. 2020;19:670–7. 10.1016/S1474-4422(20)30182-4 32702336

[36] Abdallah A Demaerschalk BM Kimweri D. A comparison of the Full Outline of Unresponsiveness (FOUR) and Glasgow Coma Scale (GCS) scores in predicting mortality among patients with reduced level of consciousness in Uganda. Neurocrit Care. 2020;32:734–41. 10.1007/s12028-019-00806-4 PMC7004860 31392656

[37] Hu X Tian J Xie J. Predictive role of shock index in the early formation of cerebral infarction in patients with TBI and cerebral herniation. Front Neurol. 2022;13:956039. 10.3389/fneur.2022.956039 PMC9454297 36090875

[38] Allison RZ Nakagawa K Hayashi M Donovan DJ Koenig MA. Derivation of a predictive score for hemorrhagic progression of cerebral contusions in moderate and severe traumatic brain injury. Neurocrit Care. 2017;26:80–6. 10.1007/s12028-016-0303-5 PMC5233574 27473209

[39] Vedantam A Yamal JM Rubin ML Robertson CS Gopinath SP. Progressive hemorrhagic injury after severe traumatic brain injury: effect of hemoglobin transfusion thresholds. J Neurosurg. 2016;125:1229–34. 10.3171/2015.11.JNS151515 PMC5065393 26943843

[40] Wang R Yang DX Ding J. Classification, risk factors, and outcomes of patients with progressive hemorrhagic injury after traumatic brain injury. BMC Neurol. 2023;23:68. 10.1186/s12883-023-03112-x PMC9926699 36782124

[41] Li Y Liu C Xiao W Song T Wang S. Incidence, risk factors, and outcomes of ventilator-associated pneumonia in traumatic brain injury: a meta-analysis. Neurocrit Care. 2020;32:272–85. 10.1007/s12028-019-00773-w PMC7223912 31300956

[42] Chang T Yan X Zhao C Zhang Y Wang B Gao L. Risk factors and neurologic outcomes in patients with traumatic brain injury and coagulopathy within 72 h after surgery. Neuropsychiatr Dis Treat. 2021;17:2905–13. 10.2147/NDT.S323897 PMC8439966 34531657

